# Intra-Species Response Variability of *Listeria monocytogenes* and *Salmonella enterica* to Lemon Essential Oils in Cheese- and Fish-Based Spreadable Foods

**DOI:** 10.3390/foods15111998

**Published:** 2026-06-03

**Authors:** Raimondo Gaglio, Antonio Alfonzo, Giuliana Garofalo, Rosa Guarcello, Valeria Guarrasi, Nicola Francesca, Giancarlo Moschetti, Luca Settanni

**Affiliations:** 1 Department of Agricultural, Food and Forest Sciences, University of Palermo, Viale Delle Scienze 4, 90128 Palermo, Italy; raimondo.gaglio@unipa.it (R.G.); antonio.alfonzo@unipa.it (A.A.); rosa.guarcello@unipa.it (R.G.); nicola.francesca@unipa.it (N.F.); giancarlo.moschetti@unipa.it (G.M.); 2 Institute of Biophysics, National Research Council, Via Ugo La Malfa 153, 90146 Palermo, Italy; valeria.guarrasi@ibf.cnr.it

**Keywords:** growth potential, lemon essential oils, *Listeria monocytogenes*, natural food preservation, salmon spreadable past, *Salmonella enterica*

## Abstract

This study examined the growth dynamics of *Salmonella enterica* and *Listeria monocytogenes* in two food matrices, cheddar cheese sauce (CCS) and salmon spreadable paste (SSP), and assessed the antimicrobial efficacy of freshly extracted essential oils (EOs) from Citrus limon cv. Femminello Santa Teresa (FST) compared with commercial (COM) EOs. Sensory sniffing tests indicated that lemon EOs were unsuitable for bio-preserving CCS. SSP supported rapid growth of both pathogens, whereas CCS caused an immediate and progressive population decline, highlighting strong matrix-dependent effects mainly related to pH. When applied to SSP, FST EOs significantly modified microbial behaviour, showing bacteriostatic activity against *S. enterica* and a rapid, irreversible bactericidal effect against *L. monocytogenes*, with complete inactivation within 24 h and no regrowth. In contrast, COM EOs showed weaker antimicrobial activity, producing limited growth reductions. SSP exhibited high growth potential (δ > 0.5) for both pathogens, with intra-species variability. FST EOs eliminated *L. monocytogenes* growth, yielding strongly negative δ values, while *Salmonella* δ values remained > 0.5, indicating reduced yet persistent growth. Throughout the experiments, pH and water activity (a_w_) remained nearly unchanged after EO addition, confirming that inhibition depended on EO bioactivity rather than matrix modification. Overall, FST lemon EOs represent a promising preservation strategy for fish spreads.

## 1. Introduction

The control of foodborne pathogens remains a major challenge for the food industry [1]. Among the most relevant bacterial hazards, *Salmonella enterica* and *Listeria monocytogenes* continue to be responsible for numerous outbreaks and product withdrawals worldwide due to their ability to survive and proliferate under a wide range of environmental conditions, including refrigeration [2,3]. Their persistence in high-moisture and protein- and fat-rich foods underscores the need for effective and consumer acceptable antimicrobial strategies [4,5]. In recent years, growing consumer demand for “clean label” products and concerns regarding synthetic preservatives have stimulated interest in natural antimicrobial compounds [6], particularly essential oils (EOs) extracted from aromatic plants and citrus fruits [7]. EOs are recognized for their broad-spectrum antimicrobial, antioxidant, and antifungal properties, making them promising candidates for incorporation into food preservation systems [8]. Citrus-derived EOs, and lemon EOs in particular, have attracted attention due to their high content of bioactive monoterpenes such as limonene, β pinene, and γ terpinene, which contribute to their antimicrobial efficacy against a variety of foodborne microorganisms [9,10].

Several studies have demonstrated the inhibitory activity of lemon EOs against pathogens including *Salmonella* spp. and *L. monocytogenes* in different food matrices and model systems. For example, emulsion-based formulations containing lemon EOs have shown significant antimicrobial effects against *Salmonella* Paratyphi A and *L. monocytogenes* [9]. Similarly, lemon EOs has been reported to reduce *L. monocytogenes* viability in meat products, highlighting its potential as a natural preservative in high-risk foods [10]. These findings support the exploration of lemon EOs as a functional ingredient capable of enhancing the microbiological safety of ready-to-eat foods.

Despite these promising results, the antimicrobial performance of EOs can vary considerably depending on the food matrix, due to interactions with lipids, proteins, and other components that may reduce their bioavailability and efficacy [11]. Therefore, evaluating EOs activity directly within real food systems is essential to determine their practical applicability. In this context, gluten-free Cheddar cheese sauce and spreadable salmon paste represent relevant matrices, as both are widely consumed, refrigerated for several days after opening, used as salad dressings, and may be potential vehicles for *Salmonella* and *L. monocytogenes* contamination.

The present study investigates the antimicrobial activity of lemon EOs, both a cultivar-specific extract (Femminello Santa Teresa) and a commercial formulation, against *S. enterica* serovars and *L. monocytogenes* strains inoculated into two different food models (an acidic cheese sauce and a salmon paste). By monitoring bacterial growth dynamics over a 14-day room-temperature storage period, this work aims to provide insight into the potential application of lemon EOs as a natural preservative in dressing sauces.

## 2. Materials and Methods

### 2.1. Raw Materials and Bacterial Strains

The essential oils (EOs) evaluated in this study were extracted from lemon (*Citrus limon* L. Burm.) fruits. Specifically, fruits of the cultivar Femminello Santa Teresa (FST) were harvested from the orchard “Parco d’Orleans” belonging to the Department of Agricultural, Food and Forest Science (SAAF), University of Palermo (Italy). This cultivar has previously demonstrated high antimicrobial efficacy against major foodborne pathogens, including *Listeria monocytogenes*, *Staphylococcus aureus*, *Salmonella enterica*, and *Enterobacter* spp. [12,13]. EOs were extracted from freshly peeled fruit flavedo by hydrodistillation using a Clevenger-type apparatus (Comandè, Palermo, Italy). Distillation was carried out for 3 h, and the EOs fraction was collected in hexane and stored in amber vials at 4 °C until use. A commercial lemon EOs (COM) was also included for comparison and was kindly provided by Eurofood s.r.l. (Capo d’Orlando, ME, Italy).

**Figure 1:**
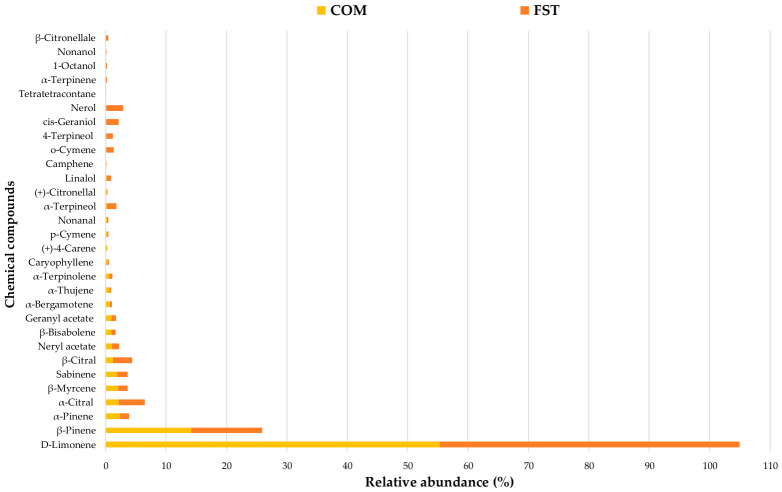
Chemical composition of Commercial (COM) and Femminello Santa Teresa (FST) essential oils.

The composition of both essential oils (COM and FST) was previously characterized by gas chromatography–mass spectrometry (GC-MS) [12] and is graphically presented in Figure 1.

The bacterial panel consisted of seven *S. enterica* serovars, *S.* Agona 50360, *S.* Derby 50399, *S.* Hadar 50272, *S.* Muenchen 50393, *S.* Enteritidis 50339 and ATCC 13076, and *S.* Newport 50404, and seven *L. monocytogenes* strains (ATCC 19114, 14BO, 133, 136, 185, 187, and 188). Except ATCC (American Type Culture Collection) coded strains, all other strains originated from the culture collection of the SAAF Department. Prior to experimentation, the isolates were reactivated and sub-cultured on Brain Heart Infusion (BHI) agar (Oxoid, Basingstoke, UK), and incubated at 37 °C for 16 h to obtain fresh cultures in the exponential growth phase.

Two food matrices were selected as model systems for subsequent assays: gluten-free Cheddar cheese sauce (Calvé^®^, Unilever Italia Mkt Operations S.r.l., Rome, Italy) and spreadable salmon paste (Rio Mare, Bolton Food s.p.a., Cermenate, Italy). These products were chosen to simulate potential vehicles for *Salmonella* and *L. monocytogenes* contamination.

### 2.2. Experimental Plan

The antimicrobial activity of the lemon EOs was assessed using the two distinct ready-to-eat food matrices gluten-free Cheddar cheese sauce (CCS) and spreadable salmon paste (SSP). Each matrix was evaluated independently to account for the potential influence of intrinsic product characteristics, such as pH, fat content, and water activity (a_w_), on bacterial behaviour and EOs efficacy. For each food model, an uninoculated sample was included as a negative control (CTR) to monitor background microbiota and ensure the absence of natural contamination throughout the experimental period.

Each of the fourteen bacterial strains (seven *Salmonella enterica* serovars and seven *L. monocytogenes*) was individually inoculated into CCS and SSP. The inoculation level was standardized to achieve an initial cell density of approximately 10^4^ colony forming units (CFU)/g, a value selected to simulate realistic massive contamination scenarios in processed foods and salad dressings. Following inoculation, three aliquots of 50 g were prepared for each strain and for the CTR samples. The aliquots were transferred into sterile 200 g plastic cups (Anicrin, Scorzé, Italy), which were then assigned to the different treatment conditions.

For each bacterial strain and food matrix, one set of cups was left untreated and served as the inoculated control. The remaining two sets were supplemented with either the FST or COM EOs, both applied at a final concentration of 1 mL/kg.

The inoculated and EO-treated samples were stored at ambient temperature to simulate a massive thermal abuse and monitored for a 14-day period. Microbiological analyses were carried out at several defined intervals: immediately after inoculation (t_0_), every 2 h during the first 8 h (t_2_ h–t_8_ h), and then every 24 h from day 1 to day 14 (t_1_ d–t_14_ d). These time points were selected to capture both the early microbial response to the EOs treatments and any subsequent regrowth or adaptation over time. The 14-day monitoring period exceeds the typical refrigerated shelf-life of products such as salad dressings and other ready-to-use foods, which are generally consumed within one week [14], and was chosen to better evaluate the behaviour of the added strains under extended storage conditions.

To ensure robustness and reproducibility of the results, the entire experimental procedure was performed in duplicate (technical replicates). Additionally, the full experiment was repeated after one month under identical conditions, generating two independent experimental replicates. In total, four replicate samples were obtained for each combination of bacterial strain, food matrix, and treatment condition.

### 2.3. Acceptability of Lemon EOs in Food Models

The sensory acceptability of lemon EOs incorporated into CCS and SSP was evaluated using COM EOs through a sniffing assay. The assessment was conducted by a trained sensory panel consisting of 15 judges (8 males and 7 females, aged 25–85 years). All panellists were familiar with the consumption of cheese and fish products, although they reported a lower habitual intake of cheese sauces and fish-based spreads. The sensory protocol was based on an empirical approach inspired by a previously published study [15] and structured according to a more recent application proposed by Garofalo et al. [16] for the evaluation of newly formulated food products. In particular, Klein and co-workers [15] described a sniffing-based sensory evaluation using a simplified scoring system to assess odour attributes. Building on this approach, the method adopted in the present study employs a descriptive scale specifically designed for preliminary acceptability screening. Samples of CCS and SSP supplemented with EOs were presented to the panel, and odour acceptability was rated using a five-point descriptive scale, where a score of 5 indicated maximum acceptability and a score of 1 corresponded to complete unacceptability due to odours inconsistent with the typical sensory profile of cheese- or fish-based products. A score of 3 was established as the minimum threshold for acceptability. Control samples consisting of CCS and SSP without EOs addition were included for comparison. The use of COM EO in this assay was intended to reflect realistic application conditions, as commercially available essential oils are more relevant for industrial use. This sensory evaluation aimed to determine whether the incorporation of lemon EOs at the tested concentration could be sensory tolerated without adversely affecting the characteristic aroma of the food matrices.

### 2.4. Microbiological Analysis

Quantification of bacterial populations in the food matrices was performed through standard serial dilution and plate count techniques. Sample preparation began by transferring 10 g of each homogenized food aliquot into sterile bags containing 90 mL of Ringer’s solution (Sigma-Aldrich, Milan, Italy). The mixtures were homogenized using a stomacher (BagMixer^®^ 400, Interscience, Saint-Nom, France) at maximum speed for 2 min to ensure thorough dispersion of microbial cells. Subsequent decimal dilutions were prepared in sterile test tubes containing 9 mL of Ringer’s solution. From each dilution, appropriate volumes were spread-plated onto selective agar media specific for the target microorganisms. Enumeration of *L. monocytogenes* was performed using *Listeria* Agar according to Ottaviani and Agosti (ALOA), with plates incubated at 37 °C for 24 h, following the guidelines of ISO 11290-2 [17]. For *Salmonella* spp., enumeration was carried out on xylose lysine desoxycholate (XLD) agar, with incubation at 37 °C for 24 h, in accordance with ISO 6579-2 [18]. All media has been purchased from Oxoid. Characteristic colonies were counted from both media and expressed as CFU/g of product.

### 2.5. Assessment of Growth Potential (δ) of L. monocytogenes and S. enterica in Spreadable Salmon

The growth potential (δ) of the 14 cultures belonging to the two pathogenic species investigated in SSP with and without lemon EOs was determined according to the approach described by Alegbeleye and Sant’Ana [19]. Growth potential was calculated as the difference between the levels (log CFU/g) measured at 144 h (six days) and those recorded immediately after inoculation (0 h). A δ value exceeding 0.5 log CFU/g was interpreted as indicative of a food system capable of supporting bacterial growth, whereas a δ value equal to or below 0.5 log CFU/g was considered representative of a system unable to support growth. Growth potential was calculated individually for each *L. monocytogenes* strain and each *S. enterica* serovar, allowing the assessment of intra-species variability in growth behavior within the SSP matrix.

### 2.6. Physicochemical Determinations

The pH of each sample was determined using a portable pH meter (Russell RL060P, Thermo Fisher Scientific, Beverly, MA, USA), previously calibrated with standard buffer solutions to ensure measurement accuracy. Water activity (a_w_) was assessed using a Rotronic Hygropalm HC2 AW meter (Rotronic AG, Bassersdorf, Switzerland). All measurements were taken after allowing the samples to equilibrate to room temperature and were conducted in duplicate.

### 2.7. Statistical Analysis

Differences among trials in sensory acceptability and microbiological datasets were evaluated by means of one-way analysis of variance (ANOVA). Tukey’s multiple comparison test was subsequently employed for pairwise comparisons, considering a significance level of *p* < 0.05. All statistical procedures were carried out using XLStat software (version 2019.2.2; Addinsoft, New York, NY, USA).

## 3. Results and Discussion

### 3.1. Acceptability of Lemon EOs in CCS and SSP

**Figure 2:**
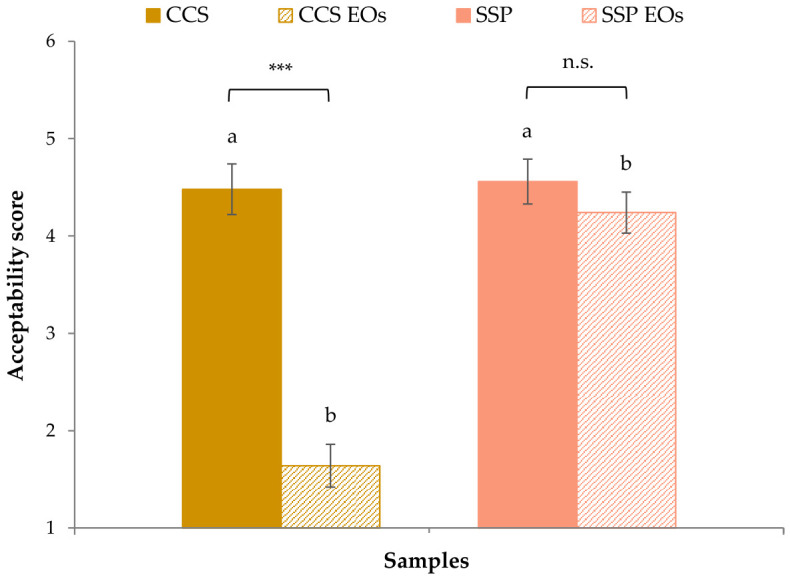
Acceptability test. Abbreviations: CCS, Cheddar cheese sauce; EOs, essential oils; SSP, spreadable salmon paste. Different lowercase letters indicate significant differences among samples. ***, *p* ≤ 0.001; n.s., not significant.

The results of the acceptability test are presented in Figure 2.

The food models without EOs addition exhibited generally high levels of acceptability for both matrices. None of the control samples reached the maximum score of 5, likely because some panellists were not fully accustomed to consuming cheese sauces or spreadable fish-based products.

The addition of lemon COM EOs markedly influenced the sensory perception of the two food models. In SSP, acceptability decreased slightly due to the pronounced lemon aroma perceived during sniffing. However, this reduction was not statistically significant, and the final acceptability score remained high (4.24 and 4.56 with and without EOs, respectively). This result is consistent with the intended formulation strategy, as citrus notes are commonly associated with fish-based products [9,20] and were generally well tolerated by the panellists. In contrast, the incorporation of EOs into CCS led to a drastic decline in sensory appreciation. The strong lemon odour was perceived as incompatible with the characteristic aroma profile of cheese sauce, resulting in a very low acceptability score (1.64), which was well below the established acceptability threshold of 3.

The sniffing approach adopted in this study, although empirical, is in line with previous literature [15,21], where simplified sensory scoring systems have been successfully applied to assess odour acceptability and discriminate sensory differences in food matrices, particularly in dairy-based products.

### 3.2. Kinetics of Growth

#### 3.2.1. Kinetics of Growth of the Selected Food-Borne Pathogens in Food Models

The behaviour of *S. enterica* and *L. monocytogenes* differed substantially between the two food matrices tested. For both species, SSP supported active bacterial growth, whereas CCS induced rapid and progressive inactivation (Figure S1). These matrix-dependent responses highlight the strong influence of physicochemical properties on bacterial physiology and survival, primarily attributable to the low pH of CCS (3.57; see Section 3.3). Taken together with the acceptability test outcomes, these results indicated that CCS was not a suitable matrix for further experimentation; consequently, all subsequent analyses were conducted using SSP.

**Figure 3:**
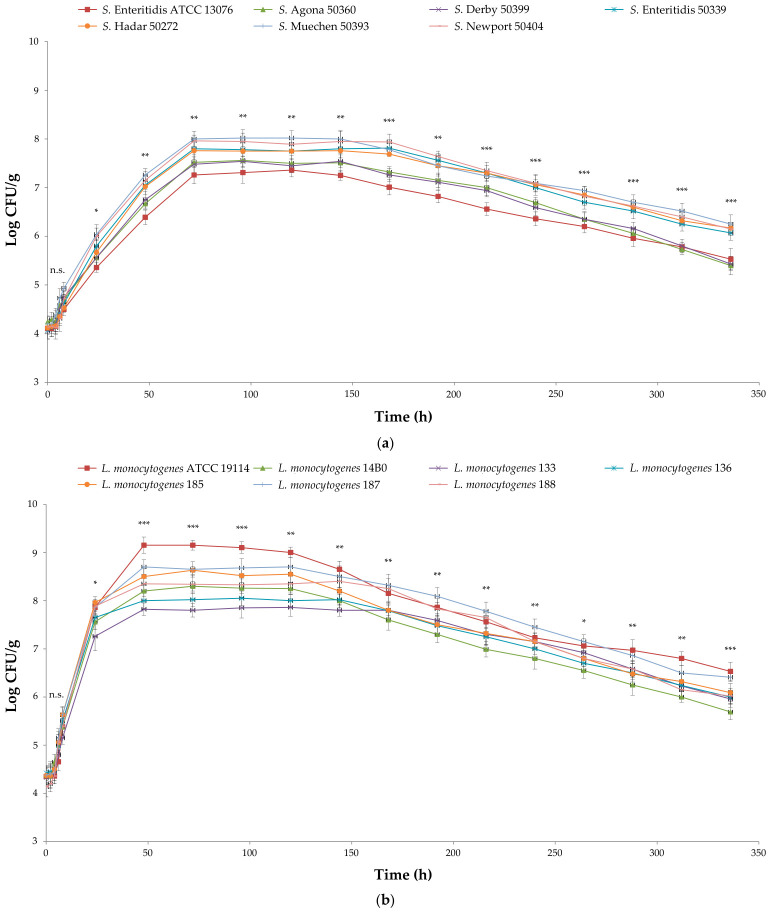
Growth kinetics of *Salmonella enterica* (**a**) and *Listeria monocytogenes* (**b**) in spreadable salmon paste. * *p* < 0.05; ** *p* < 0.01; *** *p* < 0.001; n.s., not significant (*p* > 0.05).

Both pathogens demonstrated robust proliferation in SSP (Figure 3). For *S. enterica* (Figure 3a), initial counts (ca. 4.0 log CFU/g) were followed by a short lag phase (0–8 h), after which all serovars entered exponential growth. By 24 h, populations reached 5.3–6.0 log CFU/g, and maximum densities of 7.3–8.0 log CFU/g were achieved between 72 and 96 h. Strains such as *S.* Muenchen 50393, *S.* Newport 50404 and *S.* Enteritidis 50339 showed the fastest growth, whereas the reference strain *S.* Enteritidis ATCC 13076 reached lower maxima, consistent with its lower stress tolerance documented in the literature [22].

A similar pattern was observed for *L. monocytogenes* (Figure 3b), but with even more pronounced growth. After a brief adaptation phase, all strains entered the log phase by 6–8 h, reaching 7.2–7.9 log CFU/g at 24 h. Several isolates, particularly ATCC 19114, 185, 187 and 188, continued growing to >8.5–9.0 log CFU/g by 48–72 h, exceeding the maxima observed for *Salmonella*. This outcome is expected given the ability of *L. monocytogenes* to multiply rapidly in fish-based, nutrient-rich matrices [23,24].

After reaching stationary phase, both pathogens maintained stable populations for several days before showing a slow decline of approximately 1.5–3 log units by day 14. The persistence observed here mirrors previous findings that high-protein foods support long-term survival of both species due to their high a_w_ and protective lipid/protein matrix [25,26]. In contrast to SSP, CCS did not support growth of either pathogen. Instead, both *Salmonella* and *L. monocytogenes* exhibited progressive declines from the initial inoculum (4.0–4.3 log CFU/g) within the first 24 h (Figure S1). Although *L. monocytogenes* is generally more tolerant to acidic and osmotic stresses than Salmonella, both pathogens experienced similar declining trends. Final counts were consistently lower than the starting inoculum, with reductions above 2.0–3.0 log units depending on the strain.

The strong inhibitory effect of CCS likely results from the combined impact of intrinsic hurdles, including pH below growth-permissive thresholds, cheese-derived organic acids, high fat content, which can induce oxidative and membrane stresses, salt, and emulsifying salts (e.g., citrates, phosphates) known to destabilize bacterial cell envelopes. Similar inhibitory behaviour has been reported in processed cheese products, where *Listeria* and *Salmonella* fail to grow, especially when pH is low and emulsifiers are present [27,28]. The inactivation patterns observed here are therefore consistent with the literature and reinforce the role of CCS as an unfavourable matrix for pathogen proliferation.

The combined results highlight that matrix composition was the dominant factor shaping microbial behaviour in CCS and SSP. While SSP supported rapid growth of both pathogens, although *L. monocytogenes* reached higher maxima and exhibited faster early growth, CCS imposed multi-factorial stress leading to inactivation of both species. The persistence of pathogens in SSP for more than 14 days underscores the microbiological risk associated with fish-based spreads. The strong contrast between matrices justifies the need to test the antimicrobial effectiveness of essential oils under realistic food conditions. Due to the inactivation of CCS, these tests were only conducted in SSP.

#### 3.2.2. Effect of FST EOs on the Kinetics of *S. enterica* and *L. monocytogenes* in SSP

**Figure 4:**
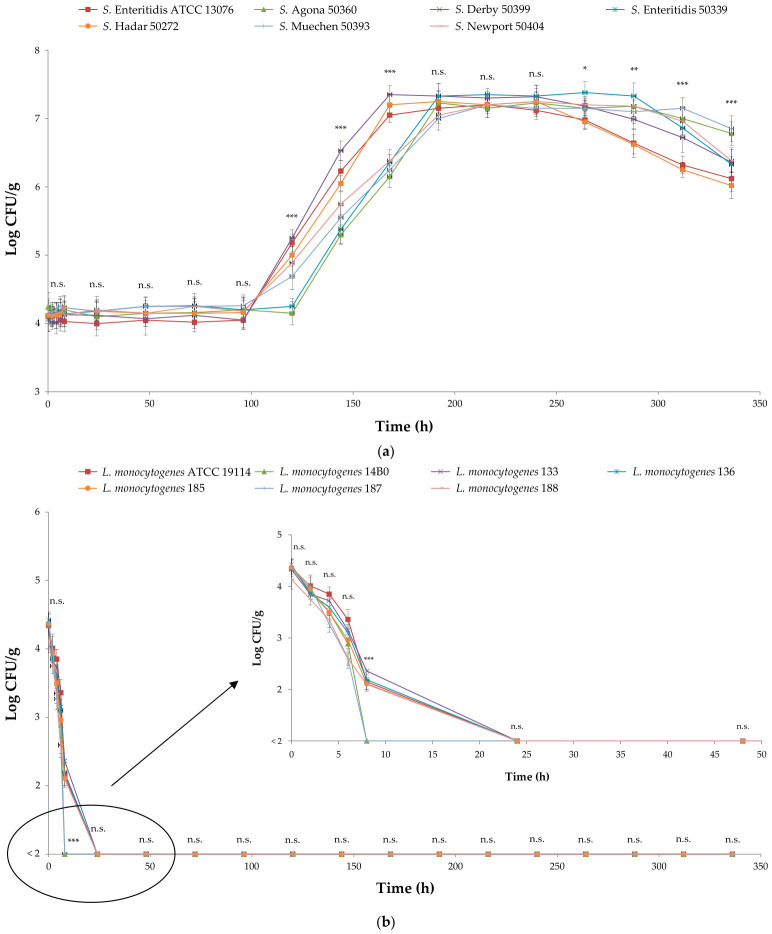
Growth kinetics of *Salmonella enterica* (**a**) and *Listeria monocytogenes* (**b**) in spreadable salmon paste added with lemon Femminello Santa Teresa (FST) essential oils. An inset in panel (**b**) shows a magnified view of the first 50 h of incubation to better highlight the bactericidal effect. * *p* < 0.05; ** *p* < 0.01; *** *p* < 0.001; n.s., not significant (*p* > 0.05).

The addition of the FST EOs markedly modified the microbial dynamics of both pathogens in SSP (Figure 4), although the magnitude and nature of inhibition differed substantially between *S. enterica* and *L. monocytogenes*. FST EOs determined bacteriostatic activity against *Salmonella* serovars whereas it exerted an almost immediate and dramatic bactericidal effect on *L. monocytogenes*.

In the FST EOs treated SSP, *Salmonella* exhibited a pronounced slowdown from the growth trends observed in the untreated matrix (Figure 4a). During the first 96 h, all serovars remained essentially unchanged around 4.0–4.2 log CFU/g, showing no evidence of growth. This plateau clearly indicates an extension of the lag phases. Only after 120 h did some strains show limited increases (e.g., *S.* Enteritidis ATCC 13076 rising to 5.18 log CFU/g and *S.* Derby reaching 5.25 log CFU/g). However, growth remained substantially below the levels recorded in untreated SSP, where the same strains exceeded 7.5–8.0 log CFU/g. At 144–168 h, populations rose moderately (5.3–7.3 log CFU/g depending on the strain), but still remained 1.5–2.0 log units lower than in the control. The following days showed stabilization and a slow decline (to 6.0–6.8 log CFU/g by day 14). These results indicate that FST EOs exerted a clear growth-delaying effect on *Salmonella*, although the magnitude of inhibition varied among serovars. In all cases, the maximum population levels remained substantially lower than those of the untreated controls, indicating only partial recovery. Notably, no strain exhibited signs of rapid adaptation or resistance development during the initial days of incubation. This pattern is consistent with a bacteriostatic mode of action, in which EOs primarily interferes with bacterial proliferation rather than inducing immediate cell death. The observed delay is likely associated with an extended lag phase, reflecting the time required for cells to recover from sublethal injury. Although growth eventually resumed, it proceeded more slowly and did not reach the levels observed in the absence of treatment. Such behavior aligns with previous studies reporting that lemon and other citrus EOs disrupt membrane integrity, interfere with quorum sensing, and impair ATP production in *Salmonella*, ultimately resulting in prolonged lag phases and reduced growth rates [29,30,31].

The application of FST EOs on *L. monocytogenes* was most effective (Figure 4b). After inoculation at almost 4.3 log CFU/g, all strains showed a steep decline already within 2–4 h (falling to 3.5–4.0 log CFU/g). By 6–8 h, populations dropped drastically, with several strains reaching 2.0–2.3 log CFU/g, and two isolates (14BO and 187) even falling below the detection limit at 8 h. By 24 h, all seven strains were undetectable, and counts remained at undetectable levels (reported as <2 log CFU/g) for the remaining 14 days. This represents a 4-log inactivation within 24 h and a stable inhibitory effect with no regrowth. Similar inactivation trends were registered with *L. monocytogenes* ATCC 19118 in minced beef meat using EOs from *Thymus capitata* [32] or with a cocktail of five *L. monocytogenes* strains inoculated onto the surface of frankfurters and treated with plant-derived β-resorcylic acid, carvacrol, and trans-cinnamaldehyde [33]. Thus, FST EOs caused rapid and irreversible death of all isolates; no strain exhibited tolerance, recovery or persistence. In this case, the application of these EOs was overwhelmingly bactericidal, not merely bacteriostatic and the response was faster and stronger than that observed for *Salmonella*. This effect aligns with known high susceptibility of *L. monocytongenes* to monoterpenes, which induce massive leakage of intracellular ions, protein denaturation, and autolytic enzyme activation in Gram-positive bacteria [34]. In particular, the FST EOs used in this study is characterized by high levels of monoterpenes such as β-pinene and, especially, D-limonene and was particularly effective in vitro against *L. monocytogenes*, *Staphylococcus aureus*, *S. enterica*, and *Enterobacter* spp. [12,13]. The FST EOs showed a bacteriostatic mode of action against *Salmonella* (partial inhibition) and a bactericidal mode of action against *L. monocytogenes* (complete and rapid inactivation). This differential susceptibility is well documented.

Gram-negative bacteria possess an outer membrane enriched in lipopolysaccharides, which reduces permeability to hydrophobic EOs [35]. In contrast, Gram-positive bacteria lack this barrier, allowing rapid diffusion of oil components into the cell membrane and cytoplasm. Thus, *Listeria* is highly vulnerable, showing rapid cell lysis even at low concentrations [36,37]. The results suggested that the FST EOs possess sufficiently high terpene bioavailability to retain antimicrobial functionality even in a challenging, high-fat, protein-rich matrix. These findings reinforce the potential application of FST EOs as an effective natural preservative in high-risk fish-based preparations. For this reason, the test was extended to commercial lemon EOs.

#### 3.2.3. Effect of Commercial EOs on the Kinetics of *S. enterica* and *L. monocytogenes* in SSP

**Figure 5:**
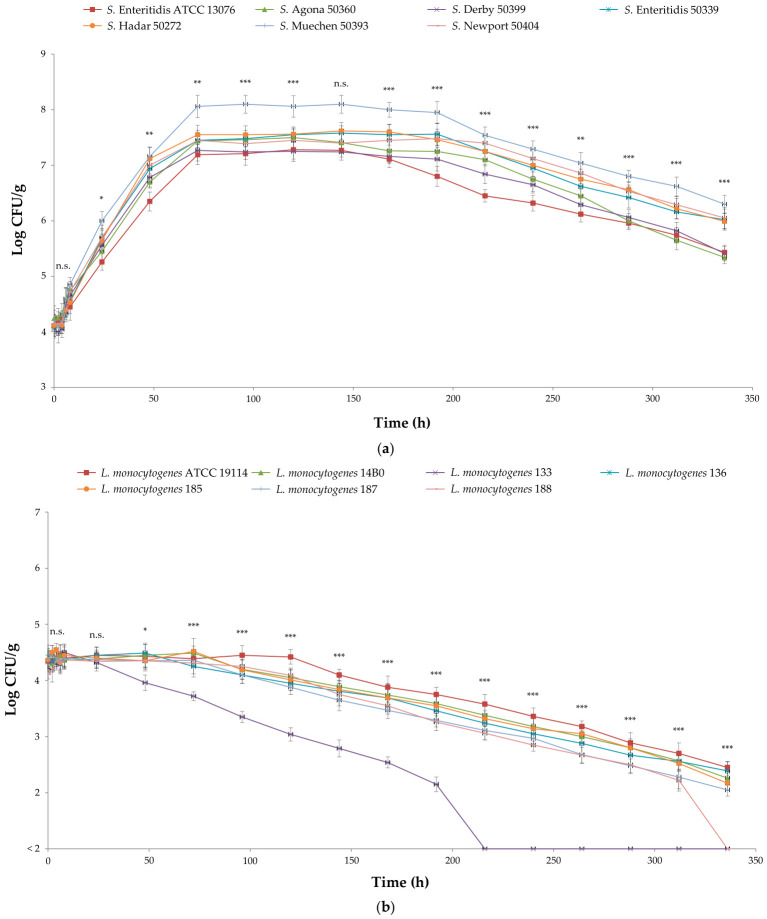
Growth kinetics of *Salmonella enterica* (**a**) and *Listeria monocytogenes* (**b**) in spreadable salmon paste added with lemon commercial (COM) essential oils. * *p* < 0.05; ** *p* < 0.01; *** *p* < 0.001; n.s., not significant (*p* > 0.05).

The commercial EOs produced antimicrobial effects notably weaker than those observed with the freshly extracted FST EOs (Figure 5). While COM EOs exerted moderate inhibition on *Salmonella* and a slow, progressive reduction in *L. monocytogenes*, it did not generate the rapid bactericidal activity observed with FST EOs. These differences reflect both the chemical variability characteristic of commercial citrus oils and the distinct physiological responses of Gram-negative and Gram-positive bacteria. In this study, the greater inhibitory activity observed for FST EOs can be attributed to its distinct chemical composition compared to COM (Figure 1). In particular, FST is richer in oxygenated monoterpenes, such as 4-terpineol, α-terpineol, cis-geraniol, β-citral, nerol, and α-citral, which are absent or present only in low amounts in the commercial lemon EOs [12].

In the COM EOs treated SSP (Figure 5a), *Salmonella* displayed growth patterns that were moderately inhibited but not suppressed. During the first 8 h, all strains increased slightly from 4.1 to 4.5 log CFU/g, which mirrors the early adaptation observed in the untreated control. Between 24 and 48 h, all seven strains entered exponential growth. By 72–120 h, populations peaked between 7.2 and 7.6 log CFU/g for most strains and 8.0 log CFU/g for *S.* Muenchen 50393 which was the highest performer. Compared with the untreated control, these maxima remain only marginally reduced, generally by 0.2–0.4 log units, indicating limited growth inhibition. After day 7, populations gradually declined, reaching 5.3–6.3 log CFU/g by day 14, following a pattern comparable to the control and significantly less pronounced than in FST EOs treated samples.

Unlike the strong bactericidal effect observed with FST EOs, COM EOs produced only a slow and progressive reduction in *L. monocytogenes* (Figure 5b). In the first 24–48 h, bacterial populations remained stable, fluctuating around 4.3–4.5 log CFU/g with minimal reductions. After 48 h, a gradual decline began, becoming more pronounced after 72–96 h. In particular, strain 133 decreased steadily to 3.35 log CFU/g at 96 h. From 120 h onward, all strains underwent a slow but consistent decline of approximately 0.2–0.4 log every 24 h.

The key efficacy differences between COM and FTS EOs are summarized in Table 1.

The reduced efficacy of COM EOs in comparison to FST EOs likely stems from a lower monoterpene content due to distillation differences, commercial blending, oxidation during storage, fraction removal (typical in industrial-grade oils), higher hydrophobic partitioning into SSP lipids which determines a reduced bioavailability, and a weaker membrane active properties, insufficient to rapidly kill Gram-positive cells [38,39]. These results clearly demonstrate that oil quality and chemical profile strongly influence antimicrobial effectiveness, and that FST represents a more promising candidate for real food preservation applications.

### 3.3. Impact of Lemon EOs on δ of L. monocytogenes and S. enterica in SSP

The results of the growth potential determination are reported in Table 2.

In the absence of EOs, both *S. enterica* and *L. monocytogenes* displayed high δ with values ranging from 3.14 to 3.81 log CFU/g for S. enterica and from 3.47 to 4.30 log CFU/g for *L. monocytogenes*. All strains largely exceeded the 0.5 log CFU/g threshold, clearly indicating that SSP is a highly permissive matrix for both pathogens. The slightly higher δ values observed for *L. monocytogenes* are consistent with its well-known ability to grow efficiently in seafood-based ready-to-eat products [40]. Notably, an intra-species variability is evident in SSP, particularly for *L. monocytogenes* (range ca. 0.8 log units), reflecting strain-dependent differences in performance and stress adaptation [41]. The addition of FST EOs resulted in markedly different responses between the two species. In case of *S. enterica* the growth potential was significantly reduced but not prevented. In fact, δ values decreased to 1.06–2.47 log CFU/g, corresponding to a reduction of approximately 1.0–2.0 log units compared to the untreated SSP. Importantly, all strains showed δ > 0.5, meaning the system remained capable of supporting growth, albeit at a slower rate. A pronounced intra-species variability emerged: *S.* Agona (δ = 1.06) and *S.* Enteritidis strains (ca. 1.3–1.4) were much more affected than other salmonellas. In contrast, FST EOs completely suppressed the growth of all *L. monocytogenes* strains, with δ values strongly negative (from −4.14 to −4.42 log CFU/g). This indicates not only an inability to support growth but an overall inactivation effect. Strikingly, the intra-species variability almost disappeared, suggesting that FST EOs exerted a uniform and dominant antimicrobial effect across *L. monocytogenes* strains.

The antimicrobial impact of COM EOs was noticeably weaker and was species-dependent. δ values (3.16–3.90 log CFU/g) were comparable to or even slightly higher those registered in SSP without EOs, indicating that COM EOs bacteriostatic effect was completely overcome in SSP. *L. monocytogenes* growth was partially inhibited, with δ values ranging from −0.25 to −1.54 log CFU/g. Data clearly showed that although growth was prevented (δ ≤ 0.5), the effect was clearly less intense than that observed with FST EOs. Within this species, the intra-species variability re-emerged, with strain 133 being more sensitive, with a δ of −1.54, than other isolates.

The application of lemon EOs revealed a species- and strain-dependent antimicrobial efficacy, with *L. monocytogenes* being markedly more sensitive than *S. enterica*. This observation is consistent with previous studies highlighting the higher susceptibility of Gram-positive bacteria to EOs than Gram-negative. The consistently high δ values (>0.5) observed across all *Salmonella* serovars indicate that, under the conditions tested, SSP remains supportive of *Salmonella* growth despite the presence of lemon EOs. In contrast, δ values suggest that SSP enriched with FST EOs may be considered unable to support the growth of *L. monocytogenes*.

### 3.4. Monitoring of pH and a_w_

The two food matrices exhibited markedly different intrinsic physicochemical properties prior to bacterial inoculation. CCS displayed a highly acidic environment with a pH of 3.57 ± 0.01, whereas SSP showed an almost neutral pH of 6.19 ± 0.02. a_w_ values followed the same pattern, with CCS at 0.950 ± 0.001 and SSP at 0.972 ± 0.001. These parameters are consistent with known characteristics of high-moisture dairy systems and protein-rich seafood emulsions, respectively, where water availability and buffering capacity differ substantially.

During the initial screening phase, CCS demonstrated complete inhibition of all 14 bacterial strains tested, including *S. enterica* and *L. monocytogenes*. The strong intrinsic antimicrobial barrier of CCS is consistent with the well-established limited growth potential of *L. monocytogenes* at low pH values approaching the lower end of its viable range (ca. pH 4.0) [42]. Likewise, *Salmonella* spp. requires a minimum a_w_ of approximately 0.93 for growth [43], a threshold slightly below the a_w_ measured in CCS. These intrinsic barriers likely account for the universal growth suppression observed, making CCS unsuitable for kinetic EOs modulation studies.

For these reasons, growth kinetics in the presence of EOs were conducted exclusively in SSP (pH 6.19, a_w_ 0.972), where both pathogens showed to be metabolically active over time.

The incorporation of lemon EOs (both COM or FST) did not induce meaningful changes in either pH or a_w_ immediately after treatment or following bacterial inoculation. This stability suggests that the oils did not interact strongly with the bulk matrix in ways that alter acidity or a_w_, an important observation in view of their food application as antimicrobial agents. EOs/food matrix interactions are known to depend on intrinsic matrix factors such as pH, lipid content, and water phase distribution, which can modulate EO solubility and antimicrobial efficacy [44]. However, in this case, SSP properties remained stable enough that pH and a_w_ were not primary variables influencing bacterial behaviour.

During the 14-day monitoring period, only minor changes in pH were observed; therefore, the corresponding graphs are not presented. Among *Salmonella* strains, the lowest average pH reached 6.08 ± 0.07, while *L. monocytogenes* exhibited a slightly greater acidification to 5.97 ± 0.06. This is consistent with reports that *L. monocytogenes* can generate acidic metabolites during growth through fermentative pathways, showing greater acidification capacity than *Salmonella*, which exhibits a more limited response under mild acid conditions [45]. The pattern for a_w_ differed from that of pH; *Salmonella* strains exhibited a more pronounced decline to 0.927 ± 0.014, whereas *L. monocytogenes* reached 0.943 ± 0.005. These shifts remain within the documented ranges permitting survival for both pathogens but approach the minimum a_w_ limit for the pathogens tested in this study. The greater a_w_ reduction associated with *Salmonella* may reflect strain-specific water-binding or matrix-interaction dynamics, while *L. monocytogenes* maintained slightly higher a_w_ values.

Importantly, the stability of pH and a_w_ in EOs treated SSP indicates that the antimicrobial effects observed in kinetic assays are attributable to the intrinsic bioactivity of the essential oils, rather than indirect effects mediated through modifications of environmental growth parameters. This is particularly critical because EOs often show reduced antimicrobial performance in real food matrices.

## 4. Conclusions

This study demonstrates that matrix composition exerts a decisive influence on pathogen behaviour, with SSP supporting prolonged growth of *S. enterica* and *L. monocytogenes*, while CCS induced rapid inactivation. Sensory evaluation unequivocally indicated that the incorporation of lemon EOs into cheese sauce was not acceptable to judges, due to a marked mismatch with its characteristic aromatic profile. Lemon FST EOs exhibited superior antimicrobial performance compared with commercial EOs, producing bacteriostatic effects against *Salmonella* and rapid bactericidal action against *L. monocytogenes*, even within a high-fat, protein-rich matrix. Accordingly, SSP supplemented with FST EOs could be classified as unable to support *L. monocytogenes* growth, as indicated by strongly negative δ values and minimal strain variability. In contrast, δ values consistently exceeding 0.5 confirmed that SSP remains supportive of *Salmonella* growth, with pronounced strain-dependent responses, highlighting persistent growth potential and the need for additional safety hurdles. Moreover, the use of lemon EOs in SSP is likely sensory acceptable, since lemon is traditionally added to seafood preparations, making its incorporation suitable for food applications from both technological and organoleptic perspectives.

A limitation of this study lies in the simplified experimental design, which did not account for the potential influence of additional food matrix components and their interactions with EOs. Therefore, further research is needed to explore these aspects under more complex and realistic conditions.

## Figures and Tables

**Table 1:** Comparative analysis of in vivo efficacy of lemon COM and FST essential oils.

Parameters	Essential Oils	
	Commercial (COM)	Femminello Santa Teresa (FST)
Effect on *Salmonella enterica*	Mild inhibition, full growth	Strong bacteriostasis (5 d), reduced maxima
Effect on *Listeria monocytogenes*	Slow decline, incomplete kill	Rapid full kill (≤24 h)
Onset of inhibition	Late (after 48–72 h)	Immediate (≤2 h)
Max reduction	2–3 log units	4 log units, permanent
Intra-species variability	High	Low

**Table 2:** Growth potential (δ) of *L. monocytogenes* and *S. enterica* in spreadable salmon paste.

Bacteria	δ (Calculated on the Average Values)		
	SSP	SSP + FST EOs	SSP + COM EOs
*S.* Enteritidis ATCC 13076	3.14	2.12	3.16
*S.* Agona 50360	3.27	1.06	3.17
*S.* Derby 50399	3.48	2.47	3.18
*S.* Hadar 50272	3.72	1.30	3.50
*S.* Muenchen 50393	3.64	1.93	3.50
*S.* Enteritidis 50339	3.80	1.35	3.90
*S.* Newport 50404	3.81	1.61	3.26
*L. monocytogenes* ATCC 19114	4.30	−4.35	−0.25
*L. monocytogenes* 14BO	3.63	−4.37	−0.48
*L. monocytogenes* 133	3.47	−4.33	−1.54
*L. monocytogenes* 136	3.60	−4.42	−0.61
*L. monocytogenes* 185	3.83	−4.37	−0.53
*L. monocytogenes* 187	4.12	−4.38	−0.73
*L. monocytogenes* 188	4.26	−4.14	−0.39

Abbreviations: SSP, spreadable salmon paste; FST, Femminello Santa Teresa cultivar; EOs, essential oils; COM, commercial; *S*., *Salmonella*; *L*., *Listeria*.

## Data Availability

The original contributions presented in this study are included in the article/Supplementary Material. Further inquiries can be directed to the corresponding authors.

## Supplementary Materials

The following supporting information can be downloaded at: https://www.mdpi.com/article/10.3390/foods15111998/s1, Figure S1: Growth kinetics of *Salmonella enterica* (a) and *Listeria monocytogenes* (b) in cheddar cheese sauce.
