# Supplementary material for: Intra-Species Response Variability of Listeria monocytogenes and Salmonella enterica to Lemon Essential Oils in Cheese- and Fish-Based Spreadable Foods
Source: Foods. 2026 Jun 3;15(11):1998. doi: 10.3390/foods15111998 (PMC13256292; doi:10.3390/foods15111998)
Supplement: Supplementary file 1 [file foods-15-01998-s001.zip › foods-4336266-supplementary.pdf]

Article

Intra-species response variability of *Listeria monocytogenes* and *Salmonella enterica* to lemon essential oils in cheese- and fish-based spreadable foods

Raimondo Gaglio [1], Antonio Alfonzo [1], Giuliana Garofalo [1,*], Rosa Guarcello [1], Valeria Guarrasi [2], Nicola Francesca [1], Giancarlo Moschetti [1], and Luca Settanni [1,*]

[1] Department of Agricultural, Food and Forest Sciences, University of Palermo, Viale delle Scienze 4, 90128 Palermo, Italy; raimondo.gaglio@unipa.it (R.Ga.); antonio.alfonzo@unipa.it (A.A.); rosa.guarcello@unipa.it (R.Gu); nicola.francesca@unipa.it (N.F.); giancarlo.moschetti@unipa.it (G.M.)

[2] Institute of Biophysics, National Research Council, Via Ugo La Malfa 153, 90146 Palermo, Italy; valeria.guarrasi@ibf.cnr.it (V.G.)

* Correspondence: giuliana.garofalo01@unipa.it (G.G.); luca.settanni@unipa .it (L.S.)

Figure S1. Growth kinetics of *Salmonella enterica* (**a**) and *Listeria monocytogenes* (**b**) in cheddar cheese sauce.

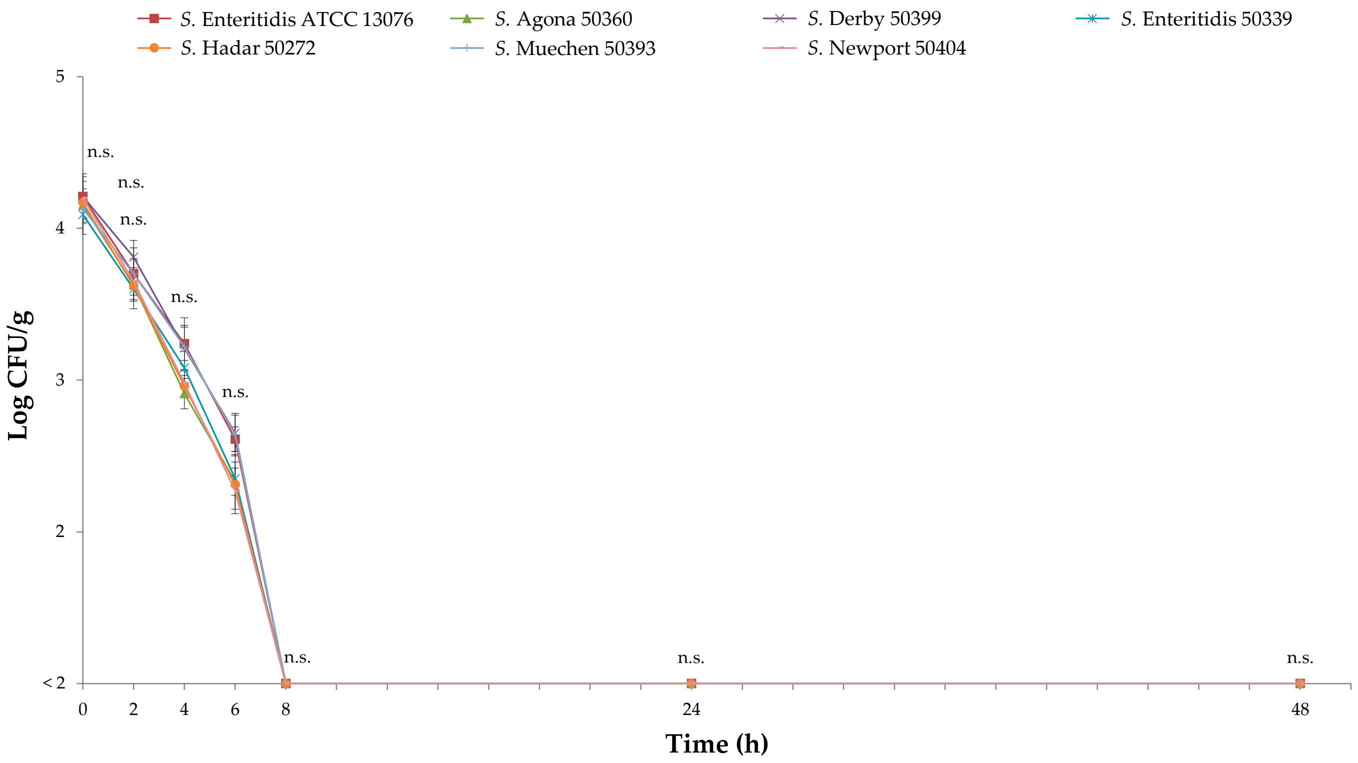

(a)

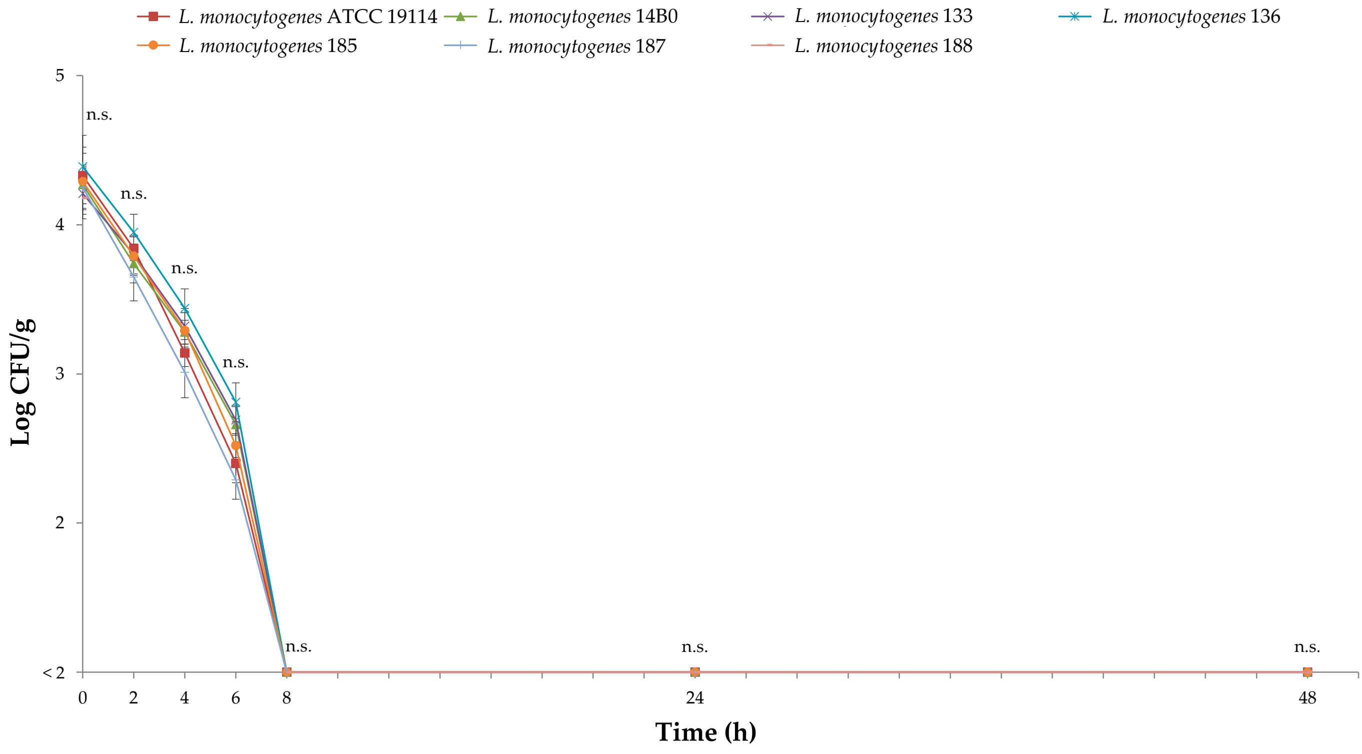

(**b**)

Figure S1. Growth kinetics of *Salmonella enterica* (**a**) and *Listeria monocytogenes* (**b**) in cheddar cheese sauce.
